# Prospective cohort study on vitamin D deficiency and its association with multiple sclerosis relapses

**DOI:** 10.6026/973206300211639

**Published:** 2025-06-30

**Authors:** Lokesh Kumar Gurram, Rohan Hassan Chandrashekara, Ashok Nimmakanty Ramadas, Sushmitha Rameshbabu, Harinishri Thambi, Sarita Sunil Chammanam, Karrar Shaaban Imran

**Affiliations:** 1 Department of Internal Medicine, University Hospital of Wales (UHW), Heath Park Way, Cardiff, CF14 4XW, UK; 2 Department of Internal Medicine, Akash Institute of Medical Sciences and Research Centre, Bengaluru, Karnataka 562110, India; 3 Department of Internal Medicine, Madras Medical College, Chennai, Tamil Nadu, India; 4 Department of Surgery, Madras Medical College, Chennai, Tamil Nadu, India; 5 Department of General Medicine, JSS Academy of Higher Education and Research, Mysuru - 570 015, Karnataka, India; 6 Department of Medicine, University of Warith Al-Anbiyaa, Karbala Governorate, Iraq

**Keywords:** Vitamin D deficiency, multiple sclerosis, ms relapses, serum 25-hydroxyvitamin d, autoimmune disease, relapse management

## Abstract

Vitamin D deficiency has been related to the pathogenesis and clinical course of multiple sclerosis (MS), especially with relapse frequency. Hence, a prospective cohort study explored the relationship between serum vitamin D level and MS relapses in 120 patients in a two-year follow-up. Baseline and follow-up serum 25-hydroxyvitamin D were measured and relapse events registered and examined. The result indicated a strong negative correlation between the frequency of relapse and vitamin D (p < 0.01), such that patients with low levels of vitamin (<20 ng/mL) had higher frequencies of relapse.

## Background:

Multiple sclerosis (MS) is a chronic autoimmune condition manifested by demyelination and neuroinflammation within the central nervous system, resulting in progressive neurological impairment [1]. The pathogenesis of MS is a combination of environmental, genetic and immunological factors [2]. Of the environmental determinants, vitamin D deficiency has proved to be one of the major risk factors for disease susceptibility and severity [3]. Vitamin D, through its immunomodulatory role, regulates T-cell function and cytokine secretion, with an important role in immune homeostasis [4]. Epidemiological data have demonstrated a greater incidence of MS in populations that have less sunlight exposure, hence suggesting vitamin D deficiency as a risk factor [5]. Levels of vitamin D have also been negatively correlated with the activity of the disease; levels have been shown to be low in association with increased relapse rates and increasing disability progression [6]. Still, the function of vitamin D in the regulation of MS relapses is poorly understood and will need further research. Therefore it is of interest to assess the relationship between serum levels of vitamin D and MS relapse rates over a two-year follow-up. Through the exploration of the relation between baseline vitamin D status and frequency of relapses, this study hopes to shed light on the possible adjunctive role of vitamin D supplementation in MS management.

## Materials and Methods:

This future cohort study was performed at a tertiary care facility for two years, recruiting 120 patients with relapsing-remitting multiple sclerosis (RRMS) according to the 2017 McDonald Criteria. Ethical clearance was received and informed consent from all participants was given. Inclusion criteria included patients aged 18-50 years with confirmed RRMS who were willing to participate in regular follow-ups and vitamin D assessments. Patients with secondary progressive or primary progressive MS, or patients on high-dose vitamin D supplementation or non-standard immunomodulatory therapies, were excluded from the study. Serum 25-hydroxyvitamin D levels at baseline were quantified and stratified into deficient (<20 ng/mL), insufficient (20-30 ng/mL), or sufficient (>30 ng/mL). A relapse was a new or exacerbating neurological sign/symptom lasting for more than 24 hours and verified clinically and radiologically. Follow-ups were conducted every three months, with vitamin D levels reassessed biannually. Data on relapses, demographic characteristics and adherence to disease-modifying therapies (DMTs) were collected and analyzed. Statistical analysis using SPSS version 25 included chi-square tests and logistic regression to evaluate the association between vitamin D levels and relapse rates, with significance set at p < 0.05.

## Results:

This study analyzed 120 patients with relapsing-remitting multiple sclerosis (RRMS) over a two-year follow-up period. The mean age of participants was 34.7 ± 7.8 years, with a female predominance (70%). Baseline serum vitamin D levels revealed 50% of patients were deficient (<20 ng/mL), 30% were insufficient (20-30 ng/mL) and 20% had sufficient levels (>30 ng/mL). Table 1 (see PDF) highlights the demographic and clinical characteristics of the study participants, showing a higher prevalence of vitamin D deficiency in females and younger patients. Table 2 (see PDF) shows the distribution of relapse rates based on vitamin D levels, revealing significantly higher relapse frequencies in the deficient group (p < 0.01). Table 3 (see PDF) depicts the relationship between seasonal variation and vitamin D levels, showing lower levels and higher relapse rates during winter months. Table 4 (see PDF) highlights the association between vitamin D supplementation and relapse rates, demonstrating a significant reduction in relapse rates among patients receiving supplementation. Table 5 (see PDF) demonstrates the relationship between relapse severity and vitamin D levels, showing that patients with deficient levels experienced more severe relapses. Table 6 (see PDF) presents relapse rates by adherence to disease-modifying therapy (DMT), showing that non-adherence was associated with significantly higher relapse rates. Table 7 (see PDF) compares the impact of vitamin D deficiency on relapse rates in patients with different baseline Expanded Disability Status Scale (EDSS) scores, showing worse outcomes in patients with higher EDSS scores. Table 8 (see PDF) highlights the correlation between vitamin D levels and MRI findings, showing a higher lesion burden in patients with deficient levels. Table 9 (see PDF) summarizes the economic impact of vitamin D deficiency, with patients in the deficient group incurring higher healthcare costs due to more frequent relapses and hospitalizations. Table 10 (see PDF) examines relapse-free survival rates, showing significantly better outcomes in patients with sufficient vitamin D levels. The study results are summarized in ten tables, depicting critical relationships between vitamin D levels and multiple sclerosis (MS) relapses. Table 1 (see PDF) details the demographic profile, which shows that the cohort has a predominance of vitamin D deficiency at 50%. Table 2 (see PDF) and Table 3 (see PDF) report higher relapse rates and lower vitamin D levels during winter. Table 4 (see PDF) demonstrates that supplementation with vitamin D significantly reduced the relapse rates. Table 5 (see PDF) and Table 6 (see PDF) show severe relapses and higher relapse rates in vitamin D-deficient patients and non-adherent DMT users. Table 7 (see PDF) highlights worse outcomes in patients with higher EDSS scores and vitamin D deficiency. Table 8 (see PDF) links deficient levels to a higher MRI lesion burden, while Table 9 reveals greater healthcare costs in deficient patients. Table 10 (see PDF) closes with favorable survival in MS free of the recurrence for people having sufficient Vitamin D, supporting its role in management as one modifiable aspect of MS care.

## Discussion:

This study highlights the significant role of vitamin D levels in influencing relapse rates and disease activity in relapsing-remitting multiple sclerosis (RRMS) [7]. A high prevalence of vitamin D deficiency (50%) was observed among the study cohort, with deficient levels strongly associated with increased relapse rates, severity and MRI lesion burden [8]. Patients with sufficient vitamin D levels had better relapse-free survival rates and lower healthcare costs, emphasizing the potential benefits of maintaining optimal vitamin D status [9]. There is a significant seasonality factor involved, with a lower level of vitamin D during winter and an increased rate of relapse; this may be a result of the reduced sunlight [10]. Vitamin D supplementation was found to significantly reduce relapse rates and thus support the use of adjunctive interventions with vitamin D supplementation [11]. The research also showed that non-adherence to DMTs increased relapse frequencies and thus the requirement for holistic management approaches [12]. Evidence available to date suggests that the level of serum vitamin D affects the risk of developing MS and also modifies disease activity in MS patients [13]. This study aligns with existing literature emphasizing the immunomodulatory effects of vitamin D and its protective role in MS. However, future research should focus on long-term randomized trials to determine optimal supplementation protocols and evaluate their impact on disease progression and patient outcomes.

## Conclusion:

A strong correlation of vitamin D deficiency with increased relapse rates in RRMS patients is shown. Low vitamin D patients had more relapses of greater severity. They also had greater lesion loads and greater healthcare utilization. In contrast, high vitamin D was seen to correlate with reduced relapse and better outcomes. These results are supportive of maintaining vitamin D in the optimal range as part of MS management. Vitamin D levels can affect disease activity and course. The inclusion of supplementation as part of management could enhance prognosis. Randomized trials in the future need to standardize dosing regimens. Additional studies should define its therapeutic potential in the treatment of MS.
